# Chiropractic Spinal Manipulation Prevents Secondary Hyperalgesia Induced by Topical Capsaicin in Healthy Individuals

**DOI:** 10.3389/fpain.2021.702429

**Published:** 2021-07-20

**Authors:** Carlos Gevers-Montoro, Benjamin Provencher, Stéphane Northon, João Paulo Stedile-Lovatel, Arantxa Ortega de Mues, Mathieu Piché

**Affiliations:** ^1^Department of Anatomy, Université du Québec à Trois-Rivières, Trois-Rivières, QC, Canada; ^2^CogNAC Research Group, Université du Québec à Trois-Rivières, Trois-Rivières, QC, Canada; ^3^Madrid College of Chiropractic, RCU Maria Cristina, Madrid, Spain

**Keywords:** manual therapy, central sensitization, back pain, pressure pain threshold, gamma band oscillations, chiropractic adjustment

## Abstract

**Background and Aims:** Spinal manipulation (SM) is currently recommended for the management of back pain. Experimental studies indicate that the hypoalgesic mechanisms of SM may rely on inhibition of segmental processes related to temporal summation of pain and, possibly, on central sensitization, although this remains unclear. The aim of this study was to determine whether experimental back pain, secondary hyperalgesia, and pain-related brain activity induced by capsaicin are decreased by segmental SM.

**Methods:** Seventy-three healthy volunteers were randomly allocated to one of four experimental groups: SM at T5 vertebral level (segmental), SM at T9 vertebral level (heterosegmental), placebo intervention at T5 vertebral level, or no intervention. Topical capsaicin was applied to the area of T5 vertebra for 40 min. After 20 min, the interventions were administered. Pressure pain thresholds (PPTs) were assessed outside the area of capsaicin application at 0 and 40 min to examine secondary hyperalgesia. Capsaicin pain intensity and unpleasantness were reported every 4 min. Frontal high-gamma oscillations were also measured with electroencephalography.

**Results:** Pain ratings and brain activity were not significantly different between groups over time (*p* > 0.5). However, PPTs were significantly decreased in the placebo and control groups (*p* < 0.01), indicative of secondary hyperalgesia, while no hyperalgesia was observed for groups receiving SM (*p* = 1.0). This effect was independent of expectations and greater than placebo for segmental (*p* < 0.01) but not heterosegmental SM (*p* = 1.0).

**Conclusions:** These results indicate that segmental SM can prevent secondary hyperalgesia, independently of expectations. This has implications for the management of back pain, particularly when central sensitization is involved.

## Background

Back pain is the leading cause of disability worldwide, entailing individual, social, and economic costs (1, 2). Every year, ~37% of the population is affected by low back pain (3). In high-income countries where the prevalence is higher (3), the economic burden has been estimated to total in the billions of dollars (1, 4, 5). In addition to the economic impact, inadequate clinical interventions can increase costs and worsen clinical outcomes (1, 6).

Current clinical practice guidelines for the treatment of back pain recommend the use of conservative interventions (7–9). These include spinal manipulation (SM), among several other manual therapies. SM is the main intervention used by chiropractors for the management of back pain (10, 11). Recent meta-analyses including individual participant data indicate that SM may be as effective as other recommended therapies for the management of chronic low back pain (12, 13). However, current data does not allow the identification of patients that will benefit more or less from SM therapy (14), in part because the mechanisms of both low back pain and its relief by SM remain unclear.

For most cases of back pain, the source of pain cannot be determined, which makes the choice of clinical intervention challenging (1, 15). When pain recurs or persists over time, it has been proposed that it is a condition in and of itself and that altered pain-related mechanisms may contribute to the disorder (16, 17). Altered pain sensitivity has been reported in patients with chronic primary low back pain (18). Central sensitization is one of the pathological processes that may contribute to altered pain sensitivity in these patients. It refers to increased spinal nociceptive transmission following sustained nociceptive inputs, which is involved in patients with chronic pain, including chronic back pain (19, 20).

Although central sensitization cannot be measured directly in humans (21), its perceptual correlates have been examined in healthy individuals using experimental pain and in patients with clinical pain (18, 22, 23). A topical application of capsaicin can evoke secondary hyperalgesia, one of the features of central sensitization that is characterized by hypersensitivity to mechanical pain stimuli beyond the area of capsaicin application (24–28). Further, capsaicin-induced pain and ongoing clinical back pain induce changes in prefrontal cortex activity (29–31). Recent findings also suggest that high-gamma oscillations can be used to examine ongoing pain-related brain processes (32–35). Thus, the assessment of secondary hyperalgesia and cerebral high-gamma oscillations could be used to evaluate the pain-relieving mechanisms of SM for back pain.

The mechanisms underlying hypoalgesia induced by SM are still largely unknown (36). SM consists of the manual application of a mechanical force on the spine, in the form of a high velocity and low amplitude thrust (37, 38). This mechanical force alters spinal biomechanics, which impacts paraspinal tissues (39–41) and sensory afferents (38, 42, 43). In turn, this initiates a cascade of neurophysiological effects that could be responsible for hypoalgesia and other clinical outcomes (38, 43, 44). It has been suggested that SM may inhibit pain through spinal segmental mechanisms, including the reduction of temporal summation during prolonged pain states (36, 45–47). Temporal summation can lead to synaptic plasticity in the spinal cord and to central sensitization (21, 48). It remains to be determined whether SM reduces central sensitization and whether this reduction underlies clinical pain relief.

The aim of the present study was to determine whether SM could reduce the development of capsaicin-induced secondary hyperalgesia and frontal high-gamma oscillations. In addition, we examined whether these effects were greater when SM was applied to the spine segments where capsaicin was applied (T5-painful area) compared with when SM was applied to spine segments without capsaicin (T9-non painful area). We hypothesized that SM would reduce capsaicin pain and secondary hyperalgesia when applied to the painful area, through segmental mechanisms. We also anticipated that SM would reduce frontal high-gamma oscillations associated with capsaicin pain.

## Methods

### Ethics Approval

All experimental procedures in this study conformed to the standards set by the latest revision of the Declaration of Helsinki and were approved by the Research Ethics Board of the Université du Québec à Trois-Rivières (Canada), as well as the Clinical Research Ethics Board of the Hospital Clínico San Carlos, Madrid (Spain). All participants gave written informed consent acknowledging their right to withdraw from the experiment without prejudice, and received a compensation of €10 for their travel expenses, time, and commitment.

### Participants

Participants were included if they were between 18 and 65 years old. They were excluded if they had been diagnosed with a physical or psychological condition, consumed alcohol regularly (>3 days per week) or on the day of the experiment, had taken any drugs during the previous 2 weeks, had a spinal surgery or physical trauma to the spine in the previous 3 months, or if they reported having an allergy/intolerance to chili peppers. One hundred and two healthy volunteers were recruited via word of mouth on the campus of the Madrid College of Chiropractic to participate in the study. Nineteen participants were included in Experiment 1 (8 women and 11 men; range 20–37 years old; mean ± SD: 22.8 ± 3.8 years old) and 83 were recruited for Experiment 2. From these 83 participants, two did not complete the experiment, resulting in the inclusion of 81 participants for Experiment 2 (40 women and 41 men; range 18–64 years old; mean ± SD: 36.5 ± 11.7 years old).

### Experimental Design: Experiment 1

Experiment 1 was a pilot study and relied on a within-subject design to characterize tonic pain produced by capsaicin applied to the back, to confirm its suitability for the main study (Experiment 2). Since capsaicin has not been used to evoke primary and secondary hyperalgesia in the back previously, the experiment aimed at identifying the time course of this experimental pain model. Participants (*n* = 19) lay prone for the entire duration of the experiment and were instructed to rate pain evoked by capsaicin for 60 min. These data were used to determine the duration of capsaicin application for Experiment 2.

### Experimental Design: Experiment 2

**Figure 1:**
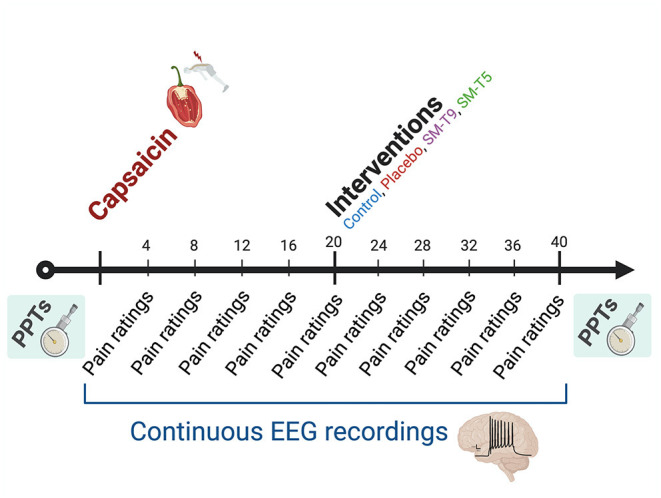
Experimental design of Experiment 2. Schematic representation of the experimental design for Experiment 2. Pressure pain thresholds (PPTs) were measured before capsaicin application and at the end of the experiment. Capsaicin was applied to the back of participants for 40 min. Pain intensity and unpleasantness were rated verbally (0–100) every 4 min and continuous brain activity was recorded with EEG. Twenty minutes after capsaicin application, the intervention was performed (placebo; spinal manipulation at T9: SM–T9; spinal manipulation at T5: SM–T5), except for the control group.

Experiment 2 relied on a mixed design to compare changes in pain perception and pain-related brain activity between four groups. A random-number generator was used to create a randomization sequence and assign participants to one of the four experimental groups: no intervention (control; *n* = 21), placebo (light mechanical stimulus applied segmentally to capsaicin pain; *n* = 20), SM applied segmentally to capsaicin pain (SM–T5; *n* = 20) and SM applied heterosegmentally to capsaicin pain (SM–T9; *n* = 20). Capsaicin was applied to the skin in the T5 vertebral segment area for 40 min while participants rated the capsaicin-evoked pain and brain activity was recorded. Pressure pain thresholds were measured in tissues surrounding the area of capsaicin application at the beginning and end of the experiment. After 20 min, the placebo, SM–T5, and SM–T9 groups received the designated intervention (see Figure 1).

### Capsaicin Pain

For both experiments, 0.6 mL of a capsaicin 1% cream (CapsiGroup, Palmira, Colombia) were applied over a 3 × 3 cm area of skin surrounding the spinous process of the T5 vertebra. This capsaicin concentration has been used to produce tonic pain in previous studies (27, 49–53). Capsaicin was uniformly distributed and pressed against the skin by applying a piece of plastic wrap over the covered region. It remained in place for 60 min in Experiment 1 and for 40 min in Experiment 2.

### Interventions

Two chiropractors performed SM. To avoid any bias that may be due to individual differences, participants were randomly assigned to one of the two chiropractors, while counterbalancing between groups. Accordingly, each chiropractor performed SM for half of the participants in both SM group. SM consisted of a short-duration, high-velocity, low-amplitude force applied to the spine to generate an audible release (cavitation). The spine was manipulated using a bilateral thenar or hypothenar contact over the transverse processes of the T5 or T9 vertebrae, depending on group allocation, after which a posterior to anterior thrust was applied to the spinal segment (47). These segments were chosen for SM to allow participants to lie prone in a stable position for the entire duration of the experiment, including the intervention period. This is necessary to allow artifact-free recording of EEG activity. A previous study showed a segmental reduction in temporal summation when SM was applied in the upper thoracic area (47). Therefore, T5 was chosen for segmental SM and T9 for heterosegmental SM. This type of manipulation typically lasts <200 ms and involves a force of ~500 Newtons (54). The placebo intervention consisted of a calibrated force of 25 N applied for 2 s on the T5 vertebral segment with a contact over the spinous process (47), using a hand-held dynamometer (model 01165, Lafayette Instrument Company, Lafayette, IN, USA). Choosing a placebo intervention for SM is challenging, as no placebo intervention can account for all aspects of SM (55). A commonly used placebo intervention consists of skin contact with no thrust, or with only soft pressing (55). The intervention aims at reproducing the SM set-up and contact with the participant. For the placebo intervention in the present study, skin contact was achieved with a hand-held dynamometer to standardize the applied force. This procedure is identical to that used in a previous study (47). In addition to the placebo group, we included a control group (no intervention) to determine if the placebo produced any effect and to measure non-specific temporal effects.

### Pain Ratings

In Experiment 1, an electronic VAS (e-VAS) consisting of a sliding transducer (Biopac Systems TSD115, Santa Barbara, CA, USA) was used to provide continuous pain intensity ratings evoked by capsaicin. Cursor position on a scale anchored at “no pain” and “worst pain imaginable” was converted to a numeric value from 0 to 100. In addition, participants were requested to rate unpleasantness verbally every 60 s using a numeric rating scale, where 0 indicated no unpleasantness and 100 indicated the worst unpleasantness imaginable. In Experiment 2, both dimensions were evaluated using verbal numeric rating scales from 0 to 100 with the same anchors. Ratings were provided every 4 min in order to limit artifacts in the EEG recordings.

In Experiment 2, before initiating the protocol for the three groups that received an intervention, participants were instructed to rate the expected change in capsaicin pain induced by the intervention. Expectations of pain relief have been shown to modulate or predict pain relief for both experimental and clinical pain (56, 57). Participants were unaware of the segmental level of SM application and that different interventions were compared between groups. The ratings were provided using a visual analog scale anchored at −100 with the descriptor “maximum pain reduction,” 0 with “no change,” and +100 with “maximum pain increase.”

### Pressure Pain Thresholds (PPTs)

In Experiment 2, in order to examine secondary hyperalgesia induced by capsaicin, pressure pain thresholds (PPTs) were evaluated at points 15 mm superior and lateral to both upper corners of the area to which capsaicin was applied, using a pressure algometer (Wagner Force Dial FDK/FDN 10, Greenwich, CT, USA) fitted with a 1 cm diameter foam pad at the end (58). Pressure was applied at a rate of ~1 kg/s, measurements were repeated twice, and threshold values were averaged (59). Participants were instructed to give a quick verbal response when pressure became painful (≥1/100). When thresholds exceeded 10 kg, the value assigned to the measurement was marked as equal to 10 kg. Thresholds were obtained before capsaicin application and at the end of the experiment, before removing the capsaicin.

### Electroencephalographic Recordings

Continuous electroencephalographic (EEG) activity was recorded at electrodes FPz, Fz, F3, F4, Cz, and Pz according to the International 10–20 system, using a linked ear lobe reference (Electro-Cap International Inc., Eaton, OH, USA). Eye movements and blinks were recorded using electro-oculographic (EOG) activity with electrodes placed at the suborbital ridge and lateral to the external canthus of the right eye. EEG and EOG were grounded with an electrode applied on the nasium and electrode impedance was kept below 10 kΩ. EEG and EOG signals were filtered using a hardware 0.1–500 Hz band-pass filter and sampled at 1,000 Hz for offline analyses.

### Electroencephalographic Analyses

Continuous EEG and EOG data were exported to MATLAB (Mathworks, Natick, MA, USA) and analyzed with EEGLAB version 14.1.0 (60). Data was down-sampled to 500 Hz and band-pass filtered (1–100 Hz) (33). A 50 Hz notch filter was set to reduce noise from external electrical sources (61). The filtered data was then re-referenced to the common average and visually inspected for infrequent and non-stereotyped artifacts (33). Finally, eye movements and muscle artifacts were removed using an independent component analysis (ICA) algorithm (62). The pre-processed data was then imported into Spike2 (Cambridge Electronic Design, Cambridge, UK) to analyze the signal from Fz as reported previously (32, 35). The continuous signal at Fz was normalized to the whole recording period (63, 64) using a Z transformation (33). The normalized EEG signal was bandpass filtered to obtain high-gamma oscillations (60–90 Hz) using a fourth-order Butterworth filter (35). The continuous recording was then transformed into the frequency domain with a Fast Fourier Transform of 512 points (33) with a Hanning window (35). High-gamma oscillation power was calculated as the area under the curve of the power spectrum from 60 to 90 Hz. This was done for each 4-min period, which included 236.1 s of data on average, after removal of artifacts. EEG data from three participants were excluded due to excessive noise (>6.5% of the time recorded, representing more than three standard deviations from the mean data rejection across participants). Rejected EEG data from the remaining participants were 1.97 % SD ± 1.51 of the total recording on average, with no significant difference between groups (*p* = 0.16). The final sample for statistical analyses consisted of 70 EEG recordings (37 women and 33 men; range 18–64 years old; mean ± SD: 36.2 ± 11.8 years old).

### Statistical Analyses

Statistical analyses were performed with Statistica v13.0 (Dell Inc., Tulsa, OK, USA). All data are expressed as mean ± SD. SD values were corrected to remove between-subject variability (65) for the repeated measures. Values of *p* ≤ 0.05 were considered statistically significant. Distribution normality was assessed with the Kolmogorov-Smirnov test and homogeneity of variance was assessed with the Levene test. Baseline measures were collected at 20 min for pain ratings (last pain rating reported before the application of the interventions) and between 16 and 20 min for gamma oscillations (last 4 min block measured before the application of the interventions). The change in pain ratings and gamma power relative to baseline was then calculated for subsequent time points and used to compare groups over time (5 time points) using Greenhouse-Geisser corrected mixed ANOVAs. Right and left PPT values were averaged and compared between groups over time (baseline vs. end of the experiment) using a Greenhouse-Geisser corrected mixed ANOVA. Significant effects were decomposed using Bonferroni-corrected planned contrasts to test a priori hypotheses (eight contrasts for changes in PPTs, and three contrasts for the effects of expectations). Effect sizes are reported based on partial eta squared ($\eta_{p}^{2}$).

## Results

### Experiment 1

#### Capsaicin Pain

**Figure 2:**
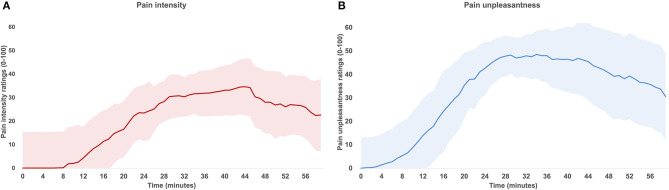
Time course of pain ratings during Experiment 1. Mean pain intensity **(A)** and unpleasantness **(B)** after capsaicin application. Both pain intensity and unpleasantness significantly increased over time (both *p* < 0.001). The shaded area represents standard deviations corrected to remove between-subject variability (see Methods).

In Experiment 1, participants reported a progressive increase in pain intensity and unpleasantness over time [*F*_(60, 180)_ = 16.8; *p* < 0.001; $\eta_{p}^{2}$ = 0.48 and *F*_(58, 1044)_ = 22.6; *p* < 0.001; $\eta_{p}^{2}$ = 0.56, respectively; see Figures 2A,B]. Between 8 and 60 min, capsaicin produced low pain intensity (mean ± SD: 20.3 ± 15.3) with a maximum of 40.1 ± 23.3. Between 2 and 60 min, capsaicin also produced low to moderate unpleasantness (mean ± SD: 31.5 ± 13.1) with a maximum of 57.6 ± 19.7. The sensation reached a plateau between 30 and 45 min after capsaicin application, from 31.1 min on average. These results were used to determine the duration of the protocol for Experiment 2 (40 min).

### Experiment 2

#### Capsaicin Pain

**Figure 3:**
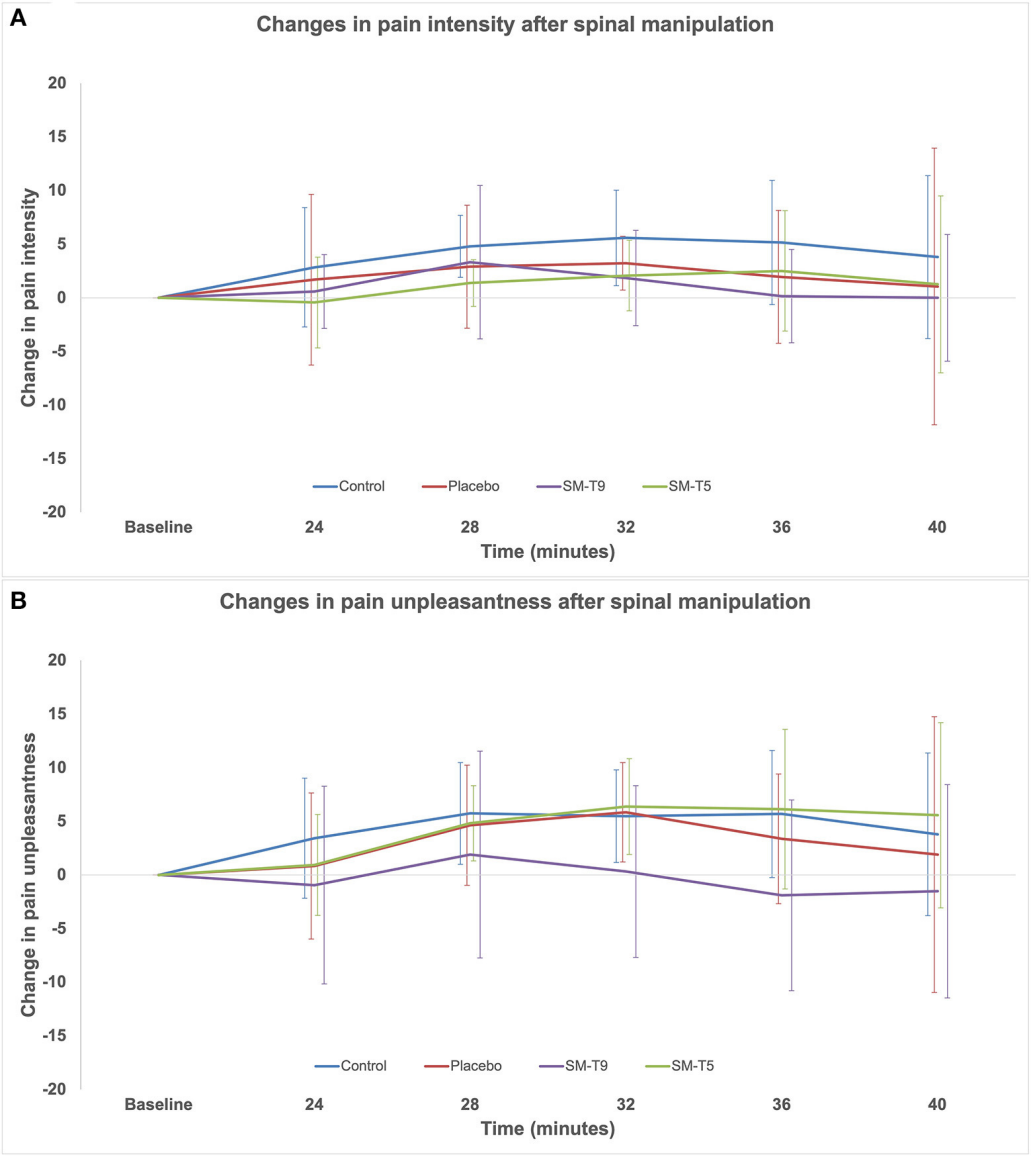
Changes in pain ratings relative to baseline in Experiment 2. Comparison of the change in capsaicin pain intensity **(A)** and unpleasantness **(B)** between groups over time, relative to baseline. Error bars represent standard deviations corrected to remove between-subject variability (see Methods). Pain intensity and unpleasantness were not significantly different between groups over time (*p* = 0.9 and *p* = 0.8, respectively). SM–T5 = spinal manipulation at T5. SM–T9 = spinal manipulation at T9.

Only the participants reporting minimum ratings of 5/100 in at least one of the two pain dimensions (intensity or unpleasantness) were included for analyses. The final sample comprised 73 participants (38 women and 35 men; range 18–64 years old; mean ± SD: 36.0 ± 11.8 years old; see Table 1 for participants' characteristics). Capsaicin pain ratings are reported for each time point during 40 min in Table 2 and the change in pain ratings from baseline are presented in Figure 3. After baseline, capsaicin pain intensity and unpleasantness did not change significantly over time for all groups combined [main effect of time: *F*_(4, 276)_ = 1.4; *p* = 0.2; $\eta_{p}^{2}$ = 0.02 and *F*_(4, 276)_ = 2.4; *p* = 0.10; $\eta_{p}^{2}$ = 0.03, respectively]. Moreover, pain intensity and unpleasantness were not significantly different between groups over time [interaction: *F*_(12, 276)_ = 0.3; *p* = 0.9; $\eta_{p}^{2}$ = 0.01 and *F*_(12, 276)_ = 0.5; *p* = 0.8; $\eta_{p}^{2}$ = 0.02, respectively].

**Table 1:** Experiment 2: characteristics of participants.

	**Control**	**Placebo**	**SM-T9**	**SM-T5**	**Total sample**
Number of participants per group	19	19	19	16	73
Sex ratio: Females/Males	10/9	9/10	10/9	9/7	38/35
Age: mean ± SD	35.5 ± 12.2	36.9 ± 9.4	37.4 ± 14.4	34.0 ± 11.2	36.0 ± 11.8
Expected change in pain: mean ± SD	–	−17.9 ± 41.5	−21.1 ± 57.4	−38.2 ± 45.5	−25.0 ± 48.7

**Table 2:** Experiment 2: pain intensity and unpleasantness ratings (mean ± SD).

		**4 min**	**8 min**	**12 min**	**16 min**	**20 min**	**24 min**	**28 min**	**32 min**	**36 min**	**40 min**
Control *n* = 19	Intensity	1.3 ± 13.1	3.7 ± 12.1	7.1 ± 8.3	10.2 ± 7.2	13.7 ± 9.7	16.5 ± 6.6	18.5 ± 8.1	19.3 ± 9.5	18.8 ± 9.4	17.5 ± 9.6
	Unpleasantness	1.3 ± 11.7	4 ± 11.8	8.2 ± 7.9	12.1 ± 8.6	17.9 ± 9.1	21.3 ± 7.0	23.6 ± 8.1	23.4 ± 8.9	23.6 ± 9.5	21.7 ± 10.2
Placebo *n* = 19	Intensity	0.3 ± 9.6	0.8 ± 9.7	2.9 ± 8.3	7.6 ± 5.4	11.4 ± 7.4	13.1 ± 10.0	14.3 ± 8.4	14.6 ± 6.4	13.3 ± 7.1	12.4 ± 12.8
	Unpleasantness	0.4 ± 11.5	4.1 ± 12.3	6.8 ± 8.7	11.9 ± 6.4	15.6 ± 6.5	16.5 ± 7.0	20 ± 8.5	21.2 ± 9.1	18.7 ± 9.0	17.3 ± 14.1
SM-T9 *n* = 20	Intensity	0.3 ± 9.3	1.1 ± 8.7	1.4 ± 8.7	6.3 ± 7.7	8.9 ± 7.7	9.5 ± 6.1	12.3 ± 11.1	10.8 ± 8.3	9.1 ± 6.4	8.9 ± 7.4
	Unpleasantness	0.9 ± 10.4	4.7 ± 12.1	7.5 ± 10.6	12.3 ± 9.4	16.5 ± 10.6	15.5 ± 10.1	18.4 ± 13.1	16.8 ± 11.1	14.6 ± 11.8	14.9 ± 12.9
SM-T5 *n* = 16	Intensity	0.3 ± 9.2	1.6 ± 8.1	3.2 ± 8.3	7.1 ± 7.1	12.2 ± 10.1	11.8 ± 5.0	13.6 ± 5.5	14.3 ± 5.9	14.7 ± 8.1	13.4 ± 10.3
	Unpleasantness	1.1 ± 14.2	3.1 ± 12.4	5.8 ± 12.3	12.4 ± 11.7	18.3 ± 9.0	19.3 ± 5.4	23.1 ± 9.4	24.7 ± 10.9	24.4 ± 13.6	23.9 ± 13.6

In order to limit a potential floor effect, the analysis was repeated with participants that reported pain ratings of 20 or more. This resulted in a sample of 46 participants (35.1 ± 11.8 years old, 46 women), with the following group allocation: control: *n* = 13, placebo: *n* = 11, SM–T5: *n* = 11, SM-T9: *n* = 11. With this sample, pain intensity, and unpleasantness did not change significantly over time [main effect: *F*_(4, 168)_ = 1.7; *p* = 0.20; $\eta_{p}^{2}$ = 0.04 and *F*_(4, 168_ = 2.4; *p* = 0.10; $\eta_{p}^{2}$ = 0.05, respectively] and the pain intensity and unpleasantness were not significantly different between groups over time [interaction: *F*_(12, 168)_ = 0.3; *p* = 0.9; $\eta_{p}^{2}$ = 0.02 and F_(12, 168)_ = 0.6; *p* = 0.7; $\eta_{p}^{2}$ = 0.04, respectively]. Thus, whether participants with light pain are included or not, results are similar.

### Secondary Hyperalgesia

**Figure 4:**
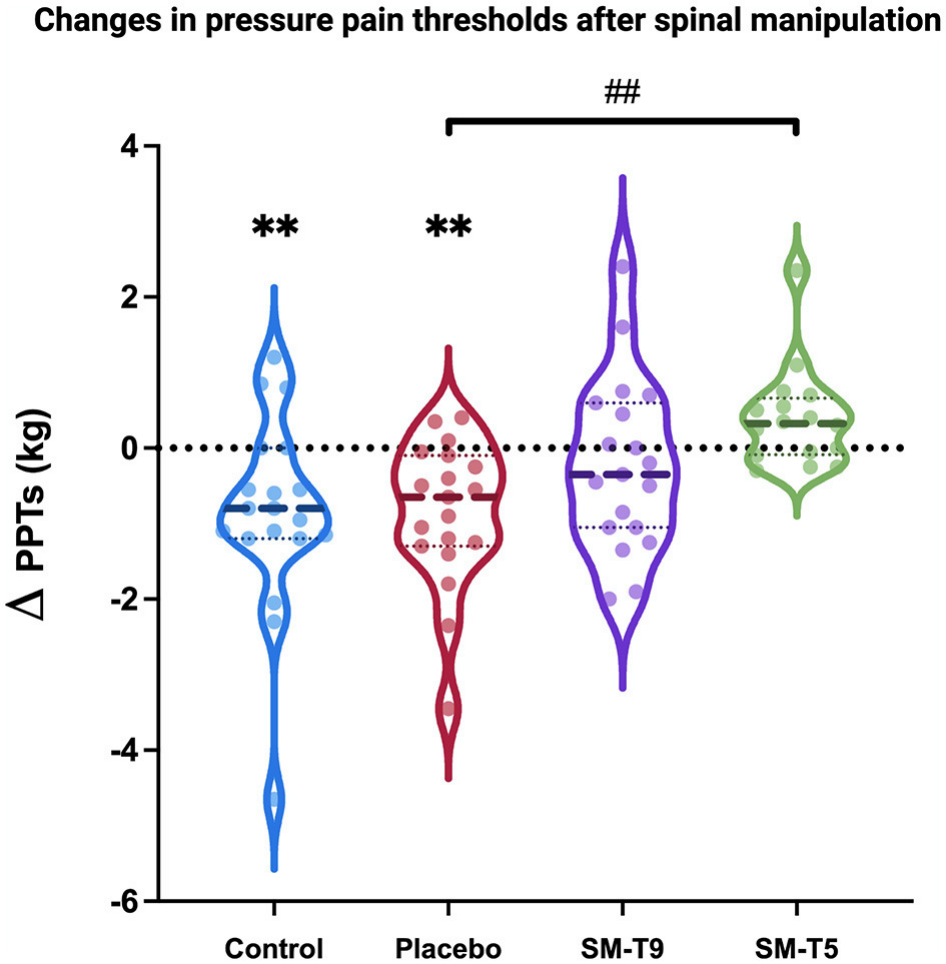
Pressure pain thresholds in Experiment 2. Comparison of changes in pressure pain thresholds (Δ_PPT_) between groups. Secondary hyperalgesia was observed in the control and placebo groups (both *p* < 0.01). SM at T5 prevented secondary hyperalgesia and the effect was significantly different compared with the placebo (*p* < 0.01). Thick dashed lines represent the median and thin dotted lines represent the 25th and 75th percentiles. SM–T5, spinal manipulation at T5. SM–T9, spinal manipulation at T9. ^**^p < 0.01, within-group; ^##^*p* < 0.01, between-group.

PPTs were significantly decreased over time [main effect: *F*_(1, 66)_ = 9.8, *p* = 0.003; $\eta_{p}^{2}$ = 0.12], and this effect was significantly different between groups [interaction: *F*_(3, 69)_ = 5.6; *p* = 0.002; $\eta_{p}^{2}$ = 0.19; see Figure 4 and Table 3]. Bonferroni-corrected planned contrasts revealed that PPTs were significantly decreased in the placebo group and the group that received no intervention (*p* = 0.005 and *p* = 0.006, respectively), indicative of secondary hyperalgesia. In contrast, no change was observed in groups that received SM at T5 (*p* = 1.0) or T9 (*p* = 1.0). Moreover, changes in PPTs were significantly different between the group that received SM at T5 and the placebo group (*p* = 0.006), indicating that SM at T5 prevented secondary hyperalgesia. However, changes in PPTs were not significantly different between the group receiving SM at T5 and the group receiving SM at T9 (*p* = 0.7). This suggests that SM at T9 produced some effects although they were not significantly different from placebo (*p* = 0.6). Lastly, the placebo group did not show significant effects compared with the group that received no intervention (*p* = 1.0).

**Table 3:** Experiment 2: mean pressure pain thresholds (mean ± SD in kg).

	**Pre**	**Post**	**Δ_(Post-Pre)_**	***p***
Control *n* = 19	5.2 ± 2.4	4.4 ± 2.1	−0.9 ± 1.3	*0.006*
Placebo *n* = 19	4.2 ± 1.7	3.4 ± 1.2	−0.9 ± 1.0	*0.005*
SM-T9 *n* = 19	4.0 ± 1.4	3.8 ± 1.7	−0.2 ± 1.1	1.0
SM-T5 *n* = 16	4.0 ± 2.1	4.4 ± 2.4	0.4 ± 0.7	1.0

*Statistically significant (< 0.05) p values are shown in Italic*.

#### Expectations

Expectations of pain relief were compared between groups (placebo, SM-T5, and SM-T9) with a one-way ANOVA. Expectations were not significantly different between groups [*F*_(2, 51)_= 0.8, *p* = 0.44, $\eta_{p}^{2}$ = 0.03; see Table 1], although the SM- T5 group expected approximately twice as much pain relief compared with the other two groups. To confirm the lack of contribution of expectations to the effect of SM on secondary hyperalgesia, a covariance analysis was conducted with PPTs from the placebo, SM-T5, and SM-T9 groups, with expectations as a covariate. This ANCOVA revealed that the decrease in PPTs over time was still significantly different between groups [interaction: *F*_(2, 51)_ = 7.5; *p* = 0.001; $\eta_{p}^{2}$ = 0.23], indicating that the group differences in secondary hyperalgesia over time were not explained by different (although not significant) expectations of pain relief between groups.

#### Brain Activity

**Figure 5:**
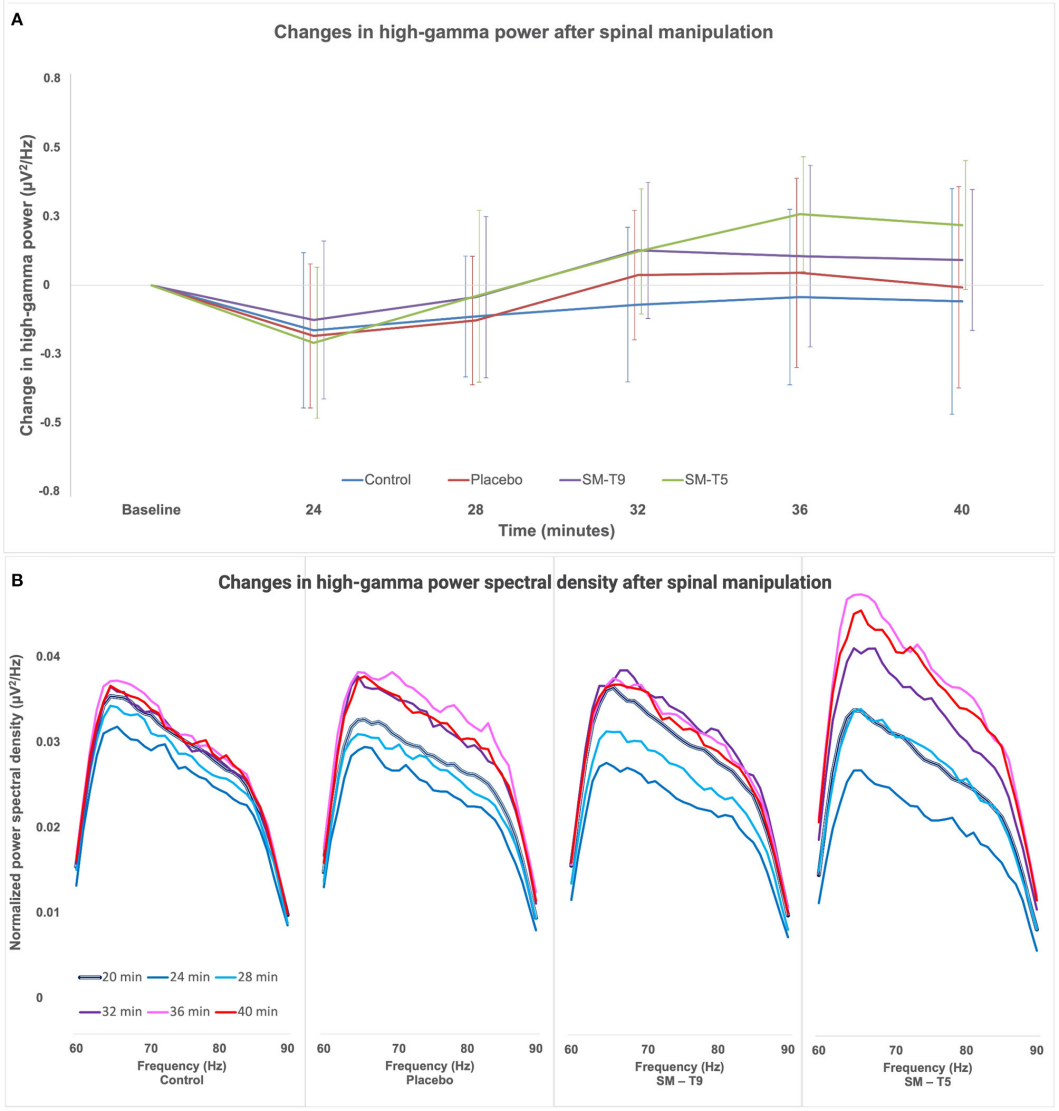
Changes in high-gamma power in Experiment 2. **(A)** Comparison of the change in high-gamma oscillation power relative to baseline between groups over time. Error bars represent standard deviations corrected to remove between-subject variability (see Methods). High-gamma power was not significantly different between groups over time (*p* = 0.5). **(B)** Changes in the power spectrum density in the high-gamma frequency range (60–90 Hz, at a definition of 0.977 Hz) relative to baseline, for the four different intervention groups. The thick black and white line represents the baseline (20 min.). Subsequent time points are represented by lines of different colors: 4 min. post-baseline (24 min. - navy-blue), 8 min. post-baseline (28 min. - light blue), 12 min. post-baseline (32 min. - purple), 16 min. post-baseline (36 min. - pink) and 20 min. post-baseline (40 min. - red). SM–T5, spinal manipulation at T5; SM–T9, spinal manipulation at T9.

High-gamma oscillation power is reported for each time point during 40 min in Table 4 and the change in high-gamma oscillation power from baseline is presented in Figure 5. High-gamma power significantly increased over time [main effect: *F*_(4, 264)_ = 9.4; *p* < 0.001; $\eta_{p}^{2}$ = 0.10], but this effect was not significantly different between groups [interaction: *F*_(12, 264)_ = 0.9; *p* = 0.5; $\eta_{p}^{2}$ = 0.04].

**Table 4:** Experiment 2: Normalized power spectral density of gamma oscillations (μV^2^/Hz).

	**4 min**	**8 min**	**12 min**	**16 min**	**20 min**	**24 min**	**28 min**	**32 min**	**36 min**	**40 min**
Control *n* = 19	0.65 ± 0.22	0.81 ± 0.35	0.99 ± 0.40	1.06 ± 0.45	0.97 ± 0.36	0.81 ± 0.24	0.86 ± 0.30	0.91 ± 0.34	0.93 ± 0.37	0.92 ± 0.45
Placebo *n* = 19	0.64 ± 0.27	0.80 ± 0.30	0.89 ± 0.32	0.96 ± 0.30	0.96 ± 0.36	0.78 ± 0.27	0.84 ± 0.23	1.00 ± 0.31	1.01 ± 0.42	0.96 ± 0.42
SM-T9 *n* = 18	0.83 ± 0.32	1.02 ± 0.38	0.93 ± 0.27	0.90 ± 0.25	0.85 ± 0.32	0.72 ± 0.24	0.80 ± 0.26	0.97 ± 0.31	0.95 ± 0.42	0.94 ± 0.35
SM-T5 *n* = 14	0.66 ± 0.32	0.82 ± 0.35	0.89 ± 0.34	0.94 ± 0.30	0.88 ± 0.24	0.68 ± 0.27	0.84 ± 0.35	1.00 ± 0.30	1.13 ± 0.24	1.09 ± 0.29

## Discussion

In the present study, topical capsaicin was applied to the back to evoke tonic pain. Spinal manipulation at the location of capsaicin-induced pain prevented the development of secondary hyperalgesia. However, capsaicin pain and frontal high-gamma oscillations were not significantly different between groups over time. The present findings suggest that SM produces anti-hyperalgesic effects that attenuate central sensitization.

### Segmental Reduction of Secondary Hyperalgesia

Pressure algometry has excellent reliability in the assessment of PPTs with an intra-class coefficient ranging between 0.8 and 0.99 (59, 67). Deep PPTs as measured in the present study are commonly used to examine changes in central sensitization (68). The results of the present study show that topical capsaicin applied to the back produces secondary hyperalgesia, as indicated by lower PPTs 15 mm outside the area of capsaicin application, in participants that received the placebo intervention or no intervention. This is consistent with previous studies that showed decreased mechanical pain thresholds 45 min to 2 h after topical capsaicin application to the volar surface of the forearm, in an area 8–10 mm beyond the area of application (26, 69).

In the SM–T5 group, SM prevented secondary hyperalgesia and this effect was significantly greater than placebo. In the SM–T9 group, SM also attenuated the development of secondary hyperalgesia, although this effect was not significantly different compared with the placebo. These results are consistent with and extend findings from a previous study that showed a reduction in the area of secondary hyperalgesia following SM, compared with a control intervention consisting of SM positioning and light manual contact (27). In this study, SM was applied at one or multiple spinal segments irrespective of the region of capsaicin application (on the forearm). These findings provide support to the hypothesis that pain relief by SM is mediated centrally, however, no specific mechanism could be inferred. By controlling for segmental and heterosegmental effects, the present study provides novel findings that indicate that secondary hyperalgesia is attenuated by SM through segmental mechanisms. Similarly, an animal study showed that ankle joint mobilization could reverse secondary hyperalgesia induced by intradermal capsaicin injection in the lateral ankle (70). Together, these findings indicate that the activation of joint and/or muscle mechanoreceptors during SM or mobilization (38) regulates central sensitization processes, likely via segmental mechanisms.

The segmental effects of SM in the present study are consistent with a large body of evidence showing that PPTs are increased segmentally after the application of SM (71–73). While previous research has focused predominantly on investigating segmental effects in non-painful segments in healthy participants, the present results indicate that SM may influence PPTs of sensitized segments. This is in line with an increase of PPTs when SM is applied to the segment with the highest pain sensitivity compared with the segment with the higher stiffness in patients with low back pain (66). However, it should be noted that in the SM–T9 group, SM also attenuated the development of secondary hyperalgesia, although the effect was not significantly different compared with placebo. This suggests that SM may also produce anti-hyperalgesic effects when applied heterosegmentally, although they may be weaker than when SM is applied to the painful segment.

In addition to the segmental mechanism underlying increased PPTs, SM-induced pain inhibition in the back or in related dermatomes was shown to depend on the inhibition of processes related to temporal summation (45, 47, 74). Repeated or sustained activation of nociceptive C-fibers is thought to be necessary for the induction of both temporal summation and secondary hyperalgesia (21, 75–77). Altogether, these results suggest that SM may regulate pain and prevent the transition from acute to chronic pain, which is associated with C-fiber activation through anti-hyperalgesic mechanisms involving the stimulation of joint and muscle receptors. This remains to be confirmed and should also be examined in patients with back pain using a series of SM interventions.

### Contribution of Expectations

In the current study, expectations of pain relief were measured at the beginning of the experiment in the three intervention groups to control for a potential contribution of expectations to the effects of SM. Participants were not told that different interventions were compared so we expected no difference in expectations between groups. Accordingly, no significant difference was observed. Nevertheless, we conducted a covariance analysis and confirmed the lack of contribution of expectations to the group difference in secondary hyperalgesia. This is consistent with previous findings that showed a C-fiber mediated hypoalgesic effect of SM independent of expectations (78, 79). It should also be noted that the placebo intervention in the present study did not produce any effect compared with no intervention, despite some expectations of pain relief, indicating that expectations did not reduce secondary hyperalgesia and that the placebo was inert.

### Capsaicin Pain

In the present study, capsaicin pain was not significantly decreased by SM. This contrasts with the significant decrease of capsaicin pain by SM reported previously (27). In this experiment, however, capsaicin was applied to the forearm and SM was delivered at multiple segments after the capsaicin was removed. These methodological differences may explain the different findings. More recently, no significant change in pain intensity or unpleasantness induced by a tonic cold stimulus was observed following SM (80). However, tonic pain was applied to the upper limb in that study, so it is not clear how these results are comparable.

It has been proposed that SM may have selective effects on pain thresholds, affecting mechanical pain sensitivity preferentially (81). The present results are consistent with this hypothesis; SM did not modulate chemically-mediated capsaicin pain but may attenuate the development of mechanical pain hypersensitivity. This suggests that the anti-hyperalgesic effects may be stronger than hypoalgesic effects or that primary hyperalgesia is not affected by SM, which may explain some discrepancies between studies (36, 82). This remains to be confirmed in future studies and the anti-hyperalgesic effects of SM should also be examined in regards to primary hyperalgesia, with the application of a mechanical stimulus to skin sensitized by capsaicin.

### Brain Activity

Consistent with the results for capsaicin pain, high-gamma power significantly increased over time, but this effect was not significantly different between groups. Navid et al. also reported no change in pain perception and in cerebral oscillations evoked by tonic pain after SM (80).

Frontal high-gamma oscillations were shown to be related to tonic experimental pain (32–34) and spontaneous clinical pain (35, 83). An association between pain ratings and high-gamma oscillation power at sensorimotor electrodes has also been reported for phasic pain stimuli (84–86). A limited number of studies have assessed whether gamma oscillations could be used as a biomarker of treatment-specific pain changes. For example, a significant reduction of pain-evoked gamma oscillations was reported after the use of Transcutaneous Electrical Nerve Stimulation (TENS) (61). However, the specific location of this brain activity was not examined, and no control condition was included to confirm the specificity of TENS effects. Nonetheless, future studies in which SM inhibits tonic pain compared with placebo may show a reduction of gamma oscillation power.

Although the lack of gamma power reduction is consistent with the lack of effects on capsaicin pain, one factor to consider in future studies is the position of participants during EEG recording. In the present study, EEG recordings were performed while subjects were in a prone position and some participants reported discomfort, which may have influenced EEG activity. Indeed, a recent study reported that prolonged cervico-facial contractions (grimaces) increase gamma oscillations at fronto-temporal electrodes (87). Thus, future studies should limit or control for muscle activity and ensure that pain-evoked activity and muscle activity can be separated. Another alternative would be to examine the suppression of alpha oscillations, which are suggested to be less sensitive to muscle artifacts (87). EEG recording with a larger number of electrodes would be essential in order to overcome these limitations and to allow the comparison of scalp topographies with previous studies.

### Limitations of This Study

Topical application of capsaicin to the back has not been used to evoke experimental pain in previous studies. Pain intensity and unpleasantness induced by capsaicin did not exceed 5/100 in eight participants (~10%). Large variability in the response to capsaicin application has been reported (88) and this should be considered in the design of future experiments. In the present study, it is possible that the low pain ratings in some participants may have limited the sensitivity to detect an inhibition of capsaicin pain and pain-related brain activity by SM.

Another point to consider in future studies is to confirm to what extent participants were blind to different interventions by asking whether they think they received a real or a sham intervention. In the present study, participants were informed that a force would be applied to their spine in the middle of the experiment, but they were unaware that different interventions were performed in different groups. Thus, participants were not asked if they thought that the intervention was real or sham.

## Conclusion

Overall, the present results indicate that segmental SM can prevent capsaicin-induced secondary hyperalgesia independently of expectations of pain relief. In contrast, spontaneous pain and frontal high-gamma oscillations induced by capsaicin were not modulated by SM. This suggests that SM may produce anti-hyperalgesic effects, which are relevant to patients with back pain in which central sensitization is involved. The anti-hyperalgesic effects of SM may also contribute to the treatment and prevention of chronic back pain, but this remains to be investigated.

## Data Availability Statement

The raw data supporting the conclusions of this article will be made available by the authors, without undue reservation.

## Ethics Statement

The studies involving human participants were reviewed and approved by Clinical Research Ethics Board of the Hospital Clínico San Carlos, Madrid, Spain and Research Ethics Board of the Université du Québec à Trois-Rivières, Trois-Rivières, Canada. The patients/participants provided their written informed consent to participate in this study.

## Author Contributions

CG-M contributed to study design, data collection, analyses and interpretation, and wrote the preliminary version of the manuscript. BP and SN contributed to data analyses, JS-L contributed to data collection. AO contributed to manuscript editing and guidance in the study design. MP contributed to study design, data analyses and interpretation, wrote the final version of the manuscript, and obtained funding for the study. All authors contributed significantly to this work and has read and approved the final version of the manuscript.

## Conflict of Interest

The authors declare that the research was conducted in the absence of any commercial or financial relationships that could be construed as a potential conflict of interest.

## References

[1] Hartvigsen J Hancock MJ Kongsted A Louw Q Ferreira ML Genevay S. What low back pain is and why we need to pay attention. Lancet. (2018) 391:2356–67. 10.1016/S0140-6736(18)30480-X 29573870

[2] GBD Collaborators Disease and Injury Incidence and Prevalence Collaborators. Global, regional, national incidence. prevalence, and years lived with disability for 354 diseases and injuries for 195 countries and territories, 1990-2017: a systematic analysis for the Global Burden of Disease Study 2017. Lancet. (2018) 392:1789–858. 10.1016/S0140-6736(18)32279-7.y 30496104 PMC6227754

[3] Hoy D Bain C Williams G March L Brooks P Blyth F. A systematic review of the global prevalence of low back pain. Arthritis Rheum. (2012) 64:2028–37. 10.1002/art.34347 22231424

[4] Walker BF Muller R Grant WD. Low back pain in Australian adults: the economic burden. Asia Pac J Public Health. (2003) 15:79–87. 10.1177/101053950301500202 15038680

[5] Alonso-Garcia M Sarria-Santamera A. The economic and social burden of low back pain in Spain: a national assessment of the economic and social impact of low back pain in Spain. Spine. (2020) 45:E1026–32. 10.1097/BRS.0000000000003476 32706566

[6] Buchbinder R Underwood M Hartvigsen J Maher CG. The lancet series call to action to reduce low value care for low back pain: an update. Pain. (2020) 161(Suppl.1):S57–64. 10.1097/j.pain.0000000000001869 33090740 PMC7434211

[7] Qaseem A Wilt TJ Mclean RM Forciea MA. Noninvasive treatments for acute, subacute, and chronic low back pain: a clinical practice guideline from the American College of Physicians. Ann Intern Med. (2017) 166:514–30. 10.7326/M16-2367 28192789

[8] Bussieres AE Stewart G Al-Zoubi F Decina P Descarreaux M Haskett D. Spinal manipulative therapy and other conservative treatments for low back pain: a guideline from the Canadian Chiropractic Guideline Initiative. J Manipulative Physiol Ther. (2018) 41:265–93. 10.1016/j.jmpt.2017.12.004 29606335

[9] Foster NE Anema JR Cherkin D Chou R Cohen SP Gross DP. Prevention and treatment of low back pain: evidence, challenges, promising directions. Lancet. (2018) 391:2368–83. 10.1016/S0140-6736(18)30489-6 29573872

[10] Hurwitz EL. Epidemiology: spinal manipulation utilization. J Electromyogr Kinesiol. (2012) 22:648–54. 10.1016/j.jelekin.2012.01.006 22289432

[11] Beliveau PJH Wong JJ Sutton DA Simon NB Bussières AE Mior SA. The chiropractic profession: a scoping review of utilization rates, reasons for seeking care, patient profiles, care provided. Chiropr Man Therap. (2017) 25:35. 10.1186/s12998-017-0165-8 29201346 PMC5698931

[12] Rubinstein SM De Zoete A Van Middelkoop M Assendelft WJJ De Boer MR Van Tulder MW. Benefits and harms of spinal manipulative therapy for the treatment of chronic low back pain: systematic review and meta-analysis of randomised controlled trials. BMJ. (2019) 364:l689. 10.1136/bmj.l689 30867144 PMC6396088

[13] De Zoete A Rubinstein S De Boer M Ostelo R Underwood M Hayden J. The effect of spinal manipulative therapy on pain relief and function in patients with chronic low back pain: an individual participant data meta-analysis. Physiotherapy. (2021) 112:121–34. 10.1016/j.physio.2021.03.006 34049207

[14] De Zoete A De Boer MR Rubinstein SM Van Tulder MW Underwood M Hayden JA. Moderators of the effect of spinal manipulative therapy on pain relief and function in patients with chronic low back pain: an individual participant data meta-analysis. Spine. (2021) 46:E505–17. 10.1097/BRS.0000000000003814 33186277 PMC7993913

[15] Vlaeyen JWS Maher CG Wiech K Van Zundert J Meloto CB Diatchenko L. Low back pain. Nat Rev Dis Primers. (2018) 4:52. 10.1038/s41572-018-0052-1 30546064

[16] Nicholas M Vlaeyen JWS Rief W Barke A Aziz Q Benoliel R. The IASP classification of chronic pain for ICD-11: chronic primary pain. Pain. (2019) 160:28–37. 10.1097/j.pain.0000000000001390 30586068

[17] Treede RD Rief W Barke A Aziz Q Bennett MI Benoliel R. Chronic pain as a symptom or a disease: the IASP classification of chronic pain for the international classification of diseases (ICD-11). Pain. (2019) 160:19–27. 10.1097/j.pain.0000000000001384 30586067

[18] Den Bandt HL Paulis WD Beckwee D Ickmans K Nijs J Voogt L. Pain mechanisms in low back pain: a systematic review with meta-analysis of mechanical quantitative sensory testing outcomes in people with nonspecific low back pain. J Orthop Sports Phys Ther. (2019) 49:698–715. 10.2519/jospt.2019.8876 31443625

[19] Woolf CJ. Central sensitization: implications for the diagnosis and treatment of pain. Pain. (2011) 152:S2–15. 10.1016/j.pain.2010.09.030 20961685 PMC3268359

[20] Nijs J George S Clauw D Fernández-De-Las-Peñas C Kosek E Ickmans K. Central sensitisation in chronic pain conditions: latest discoveries and their potential for precision medicine. Lancet Rheumatol. (2021) 3:E383–92. 10.1016/S2665-9913(21)00032-1 38279393

[21] Latremoliere A Woolf CJ. Central sensitization: a generator of pain hypersensitivity by central neural plasticity. J Pain. (2009) 10:895–926. 10.1016/j.jpain.2009.06.012 19712899 PMC2750819

[22] Sanzarello I Merlini L Rosa MA Perrone M Frugiuele J Borghi R. Central sensitization in chronic low back pain: a narrative review. J Back Musculoskelet Rehabil. (2016) 29:625–33. 10.3233/BMR-160685 27062464

[23] Starkweather AR Heineman A Storey S Rubia G Lyon DE Greenspan J. Methods to measure peripheral and central sensitization using quantitative sensory testing: a focus on individuals with low back pain. Appl Nurs Res. (2016) 29:237–41. 10.1016/j.apnr.2015.03.013 26856520

[24] Ali Z Meyer RA Campbell JN. Secondary hyperalgesia to mechanical but not heat stimuli following a capsaicin injection in hairy skin. Pain. (1996) 68:401–11. 10.1016/S0304-3959(96)03199-5 9121830

[25] Morris VH Cruwys SC Kidd BL. Characterisation of capsaicin-induced mechanical hyperalgesia as a marker for altered nociceptive processing in patients with rheumatoid arthritis. Pain. (1997) 71:179–86. 10.1016/S0304-3959(97)03361-7 9211479

[26] Andrews K Baranowski A Kinnman E. Sensory threshold changes without initial pain or alterations in cutaneous blood flow, in the area of secondary hyperalgesia caused by topical application of capsaicin in humans. Neurosci Lett. (1999) 266:45–8. 10.1016/S0304-3940(99)00248-7 10336180

[27] Mohammadian P Gonsalves A Tsai C Hummel T Carpenter T. Areas of capsaicin-induced secondary hyperalgesia and allodynia are reduced by a single chiropractic adjustment: a preliminary study. J Manipulative Physiol Ther. (2004) 27:381–7. 10.1016/j.jmpt.2004.05.002 15319760

[28] Quesada C Kostenko A Ho I Leone C Nochi Z Stouffs A. Human surrogate models of central sensitization: a critical review and practical guide. Eur J Pain. (2021) 1–40. 10.1002/ejp.1768 33759294 PMC8360051

[29] Baron R Baron Y Disbrow E Roberts TP. Brain processing of capsaicin-induced secondary hyperalgesia: a functional MRI study. Neurology. (1999) 53:548–57. 10.1212/WNL.53.3.548 10449119

[30] Apkarian AV Sosa Y Sonty S Levy RM Harden RN Parrish TB. Chronic back pain is associated with decreased prefrontal and thalamic gray matter density. J Neurosci. (2004) 24:10410–5. 10.1523/JNEUROSCI.2541-04.2004 15548656 PMC6730296

[31] Baliki MN Baria AT Apkarian AV. The cortical rhythms of chronic back pain. J Neurosci. (2011) 31:13981–90. 10.1523/JNEUROSCI.1984-11.2011 21957259 PMC3214084

[32] Schulz E May ES Postorino M Tiemann L Nickel MM Witkovsky V. Prefrontal gamma oscillations encode tonic pain in humans. Cereb Cortex. (2015) 25:4407–14. 10.1093/cercor/bhv043 25754338 PMC4816790

[33] Li L Liu X Cai C Yang Y Li D Xiao L. Changes of gamma-band oscillatory activity to tonic muscle pain. Neurosci Lett. (2016) 627:126–31. 10.1016/j.neulet.2016.05.067 27250858

[34] Nickel MM May ES Tiemann L Schmidt P Postorino M Ta Dinh S. Brain oscillations differentially encode noxious stimulus intensity and pain intensity. Neuroimage. (2017) 148:141–7. 10.1016/j.neuroimage.2017.01.011 28069543 PMC5349759

[35] May ES Nickel MM Ta Dinh S Tiemann L Heitmann H Voth I. Prefrontal gamma oscillations reflect ongoing pain intensity in chronic back pain patients. Hum Brain Mapp. (2019) 40:293–305. 10.1002/hbm.24373 30260531 PMC6585682

[36] Gevers-Montoro C Provencher B Descarreaux M Ortega De Mues A Piche M. Neurophysiological mechanisms of chiropractic spinal manipulation for spine pain. Eur J Pain. (2021) 1–20. 10.1002/ejp.1773 33786932

[37] Herzog W. The biomechanics of spinal manipulation. J Bodyw Mov Ther. (2010) 14:280–6. 10.1016/j.jbmt.2010.03.004 20538226

[38] Pickar JG Bolton PS. Spinal manipulative therapy and somatosensory activation. J Electromyogr Kinesiol. (2012) 22:785–94. 10.1016/j.jelekin.2012.01.015 22349622 PMC3399029

[39] Nougarou F Dugas C Deslauriers C Page I Descarreaux M. Physiological responses to spinal manipulation therapy: investigation of the relationship between electromyographic responses and peak force. J Manipulative Physiol Ther. (2013) 36:557–63. 10.1016/j.jmpt.2013.08.006 24161387

[40] Reed WR Long CR Kawchuk GN Pickar JG. Neural responses to the mechanical parameters of a high-velocity, low-amplitude spinal manipulation: effect of preload parameters. J Manipulative Physiol Ther. (2014) 37:68–78. 10.1016/j.jmpt.2013.12.004 24387888 PMC3946664

[41] Funabashi M Nougarou F Descarreaux M Prasad N Kawchuk GN. Spinal tissue loading created by different methods of spinal manipulative therapy application. Spine. (2017) 42:635–43. 10.1097/B.R. S.0000000000002096 28146021 PMC5407629

[42] Bialosky JE Bishop MD Price DD Robinson ME George SZ. The mechanisms of manual therapy in the treatment of musculoskeletal pain: a comprehensive model. Man Ther. (2009) 14:531–8. 10.1016/j.math.2008.09.001 19027342 PMC2775050

[43] Bialosky JE Beneciuk JM Bishop MD Coronado RA Penza CW Simon CB. Unraveling the mechanisms of manual therapy: modeling an approach. J Orthop Sports Phys Ther. (2018) 48:8–18. 10.2519/jospt.2018.7476 29034802

[44] Gyer G Michael J Inklebarger J Tedla JS. Spinal manipulation therapy: is it all about the brain? A current review of the neurophysiological effects of manipulation. J Integr Med. (2019) 17:328–37. 10.1016/j.joim.2019.05.004 31105036

[45] Bialosky JE Bishop MD Robinson ME Zeppieri G Jr George SZ. Spinal manipulative therapy has an immediate effect on thermal pain sensitivity in people with low back pain: a randomized controlled trial. Phys Ther. (2009) 89:1292–303. 10.2522/ptj.20090058 19797305 PMC2794479

[46] Bishop MD Beneciuk JM George SZ. Immediate reduction in temporal sensory summation after thoracic spinal manipulation. Spine J. (2011) 11:440–6. 10.1016/j.spinee.2011.03.001 21463970 PMC3092807

[47] Randoll C Gagnon-Normandin V Tessier J Bois S Rustamov N O'shaughnessy J. The mechanism of back pain relief by spinal manipulation relies on decreased temporal summation of pain. Neuroscience. (2017) 349:220–8. 10.1016/j.neuroscience.2017.03.006 28288900

[48] Woolf CJ. Evidence for a central component of post-injury pain hypersensitivity. Nature. (1983) 306:686–8. 10.1038/306686a0 6656869

[49] Domnick C Hauck M Casey KL Engel AK Lorenz J. C-fiber-related EEG-oscillations induced by laser radiant heat stimulation of capsaicin-treated skin. J Pain Res. (2009) 2:49–56. 10.2147/JPR.S4860 21197293 PMC3004625

[50] Hullemann P Watfeh R Shao YQ Nerdal A Binder A Baron R. Peripheral sensitization reduces laser-evoked potential habituation. Neurophysiol Clin. (2015) 45:457–67. 10.1016/j.neucli.2015.10.088 26602971

[51] Martel M Harvey MP Houde F Balg F Goffaux P Leonard G. Unravelling the effect of experimental pain on the corticomotor system using transcranial magnetic stimulation and electroencephalography. Exp Brain Res. (2017) 235:1223–31. 10.1007/s00221-017-4880-0 28188330 PMC5348561

[52] Schaffler K Nicolas LB Borta A Brand T Reitmeir P Roebling R. Investigation of the predictive validity of laser-EPs in normal, UVB-inflamed and capsaicin-irritated skin with four analgesic compounds in healthy volunteers. Br J Clin Pharmacol. (2017) 83:1424–35. 10.1111/bcp.13247 28139023 PMC5465336

[53] Ferland CE Villemure C Michon PE Gandhi W Ma L Chouchou F. Multicenter assessment of quantitative sensory testing (QST) for the detection of neuropathic-like pain responses using the topical capsaicin model. Can J Pain. (2018) 2:266–79. 10.1080/24740527.2018.1525682 35005384 PMC8730652

[54] Triano JJ Giuliano D Kanga I Starmer D Brazeau J Screaton CE. Consistency and malleability of manipulation performance in experienced clinicians: a pre-post experimental design. J Manipulative Physiol Ther. (2015) 38:407–15. 10.1016/j.jmpt.2015.05.002 26198595

[55] Puhl AA Reinhart CJ Doan JB Vernon H. The quality of placebos used in randomized, controlled trials of lumbar and pelvic joint thrust manipulation-a systematic review. Spine J. (2017) 17:445–56. 10.1016/j.spinee.2016.11.003 27888138

[56] Cormier S Piche M Rainville P. Expectations modulate heterotopic noxious counter-stimulation analgesia. J Pain. (2013) 14:114–25. 10.1016/j.jpain.2012.10.006 23260452

[57] Cormier S Lavigne GL Choiniere M Rainville P. Expectations predict chronic pain treatment outcomes. Pain. (2016) 157:329–38. 10.1097/j.pain.0000000000000379 26447703

[58] Hughes SW Zhao H Auvinet EJ Strutton PH. Attenuation of capsaicin-induced ongoing pain and secondary hyperalgesia during exposure to an immersive virtual reality environment. Pain Rep. (2019) 4:e790. 10.1097/PR9.0000000000000790 31984295 PMC6903343

[59] Balaguier R Madeleine P Vuillerme N. Is one trial sufficient to obtain excellent pressure pain threshold reliability in the low back of asymptomatic individuals? A test-retest study. PLoS ONE. (2016) 11:e0160866. 10.1371/journal.pone.0160866 27513474 PMC4981327

[60] Delorme A Makeig S. EEGLAB: an open source toolbox for analysis of single-trial EEG dynamics including independent component analysis. J Neurosci Methods. (2004) 134:9–21. 10.1016/j.jneumeth.2003.10.009 15102499

[61] Ebrahimian M Razeghi M Zamani A Bagheri Z Rastegar K Motealleh A. Does high frequency transcutaneous electrical nerve stimulation (TENS) affect EEG gamma band activity? J Biomed Phys Eng. (2018) 8:271–80. 10.31661/jbpe.v8i3Sep.780 30320031 PMC6169118

[62] Jung TP Makeig S Humphries C Lee TW McKeown MJ Iragui V. Removing electroencephalographic artifacts by blind source separation. Psychophysiology. (2000) 37:163–78. 10.1111/1469-8986.3720163 10731767

[63] Ellmore TM Ng K Reichert CP. Early and late components of EEG delay activity correlate differently with scene working memory performance. PLoS ONE. (2017) 12:e0186072. 10.1371/journal.pone.0186072 29016657 PMC5634640

[64] Alday PM. How much baseline correction do we need in ERP research? extended GLM model can replace baseline correction while lifting its limits. Psychophysiology. (2019) 56:e13451. 10.1111/psyp.13451 31403187

[65] Cousineau D. Confidence intervals in within-subject designs: a simpler solution to Loftus and Masson's method. Tutorials Quantitative Methods Psychol. (2005) 1:42–5. 10.20982/tqmp.01.1.p042

[66] Nim CG Kawchuk GN Schiottz-Christensen B O'neill S. The effect on clinical outcomes when targeting spinal manipulation at stiffness or pain sensitivity: a randomized trial. Sci Rep. (2020) 10:14615. 10.1038/s41598-020-71557-y 32884045 PMC7471938

[67] Mailloux C Beaulieu LD Wideman TH Masse-Alarie H. Within-session test-retest reliability of pressure pain threshold and mechanical temporal summation in healthy subjects. PLoS ONE. (2021) 16:e0245278. 10.1371/journal.pone.0245278 33434233 PMC7802960

[68] Middlebrook N Rushton AB Abichandani D Kuithan P Heneghan NR Falla D. Measures of central sensitization and their measurement properties in musculoskeletal trauma: a systematic review. Eur J Pain. (2021) 25:71–87. 10.1002/ejp.1670 33034137

[69] Zheng Z Gibson SJ Khalil Z Helme RD Mcmeeken JM. Age-related differences in the time course of capsaicin-induced hyperalgesia. Pain. (2000) 85:51–8. 10.1016/S0304-3959(99)00247-X 10692602

[70] Sluka KA Wright A. Knee joint mobilization reduces secondary mechanical hyperalgesia induced by capsaicin injection into the ankle joint. Eur J Pain. (2001) 5:81–7. 10.1053/eujp.2000.0223 11394925

[71] Coronado RA Gay CW Bialosky JE Carnaby GD Bishop MD George SZ. Changes in pain sensitivity following spinal manipulation: a systematic review and meta-analysis. J Electromyogr Kinesiol. (2012) 22:752–67. 10.1016/j.jelekin.2011.12.013 22296867 PMC3349049

[72] Millan M Leboeuf-Yde C Budgell B Amorim MA. The effect of spinal manipulative therapy on experimentally induced pain: a systematic literature review. Chiropr Man Therap. (2012) 20:26. 10.1186/2045-709X-20-26 22883534 PMC3527169

[73] Honore M Leboeuf-Yde C Gagey O. The regional effect of spinal manipulation on the pressure pain threshold in asymptomatic subjects: a systematic literature review. Chiropr Man Therap. (2018) 26:11. 10.1186/s12998-018-0181-3 29713457 PMC5907416

[74] George SZ Bishop MD Bialosky JE Zeppieri G Jr Robinson ME. Immediate effects of spinal manipulation on thermal pain sensitivity: an experimental study. BMC Musculoskelet Disord. (2006) 7:68. 10.1186/1471-2474-7-68 16911795 PMC1578563

[75] Price DD Hu JW Dubner R Gracely RH. Peripheral suppression of first pain and central summation of second pain evoked by noxious heat pulses. Pain. (1977) 3:57–68. 10.1016/0304-3959(77)90035-5 876667

[76] Torebjork HE Lundberg LE Lamotte RH. Central changes in processing of mechanoreceptive input in capsaicin-induced secondary hyperalgesia in humans. J Physiol. (1992) 448:765–80. 10.1113/jphysiol.1992.sp019069 1593489 PMC1176227

[77] Ziegler EA Magerl W Meyer RA Treede RD. Secondary hyperalgesia to punctate mechanical stimuli. central sensitization to A-fibre nociceptor input. Brain. (1999) 122 (Pt 12):2245–57. 10.1093/brain/122.12.2245 10581220

[78] Bialosky JE Bishop MD Robinson ME Barabas JA George SZ. The influence of expectation on spinal manipulation induced hypoalgesia: an experimental study in normal subjects. BMC Musculoskelet Disord. (2008) 9:19. 10.1186/1471-2474-9-19 18267029 PMC2270829

[79] Bialosky JE George SZ Horn ME Price DD Staud R Robinson ME. Spinal manipulative therapy-specific changes in pain sensitivity in individuals with low back pain (NCT01168999). J Pain. (2014) 15:136–48. 10.1016/j.jpain.2013.10.005 24361109 PMC3946602

[80] Navid MS Lelic D Niazi IK Holt K Mark EB Drewes AM. The effects of chiropractic spinal manipulation on central processing of tonic pain - a pilot study using standardized low-resolution brain electromagnetic tomography (sLORETA). Sci Rep. (2019) 9:6925. 10.1038/s41598-019-42984-3 31061511 PMC6502880

[81] Aspinall SL Leboeuf-Yde C Etherington SJ Walker BF. Manipulation-induced hypoalgesia in musculoskeletal pain populations: a systematic critical review and meta-analysis. Chiropr Man Therap. (2019) 27:7. 10.1186/s12998-018-0226-7 30719281 PMC6350309

[82] Aspinall SL Jacques A Leboeuf-Yde C Etherington SJ Walker BF. No difference in pressure pain threshold and temporal summation after lumbar spinal manipulation compared to sham: a randomised controlled trial in adults with low back pain. Musculoskelet Sci Pract. (2019) 43:18–25. 10.1016/j.msksp.2019.05.011 31176287

[83] Lim M Kim JS Kim DJ Chung CK. Increased low- and high-frequency oscillatory activity in the prefrontal cortex of Fibromyalgia Patients. Front Hum Neurosci. (2016) 10:111. 10.3389/fnhum.2016.00111 27014041 PMC4789463

[84] Gross J Schnitzler A Timmermann L Ploner M. Gamma Oscillations in Human Primary Somatosensory Cortex Reflect Pain Perception. PLoS Bio. (2001) 5:e133. 10.1371/journal.pbio.0050133 17456008 PMC1854914

[85] Zhang ZG Hu L Hung YS Mouraux A Iannetti GD. Gamma-band oscillations in the primary somatosensory cortex-a direct and obligatory correlate of subjective pain intensity. J Neurosci. (2012) 32:7429–38. 10.1523/JNEUROSCI.5877-11.2012 22649223 PMC6703598

[86] Rossiter HE Worthen SF Witton C Hall SD Furlong PL. Gamma oscillatory amplitude encodes stimulus intensity in primary somatosensory cortex. Front Hum Neurosci. (2013) 7:362. 10.3389/fnhum.2013.00362 23874282 PMC3711008

[87] Chouchou F Perchet C Garcia-Larrea L. EEG changes reflecting pain: is alpha suppression better than gamma enhancement? Neurophysiol Clin. (2021) 51:209–18. 10.1016/j.neucli.2021.03.001 33741256

[88] Liu M Max MB Robinovitz E Gracely RH Bennett GJ. The human capsaicin model of allodynia and hyperalgesia: sources of variability and methods for reduction. J Pain Symptom Manage. (1998) 16:10–20. 10.1016/S0885-3924(98)00026-8 9707653

